# Autophagy in the light of sphingolipid metabolism

**DOI:** 10.1007/s10495-015-1108-2

**Published:** 2015-02-15

**Authors:** Eva Bang Harvald, Anne Sofie Braun Olsen, Nils J. Færgeman

**Affiliations:** Villum Center for Bioanalytical Sciences, Department of Biochemistry and Molecular Biology, University of Southern Denmark, Campusvej 55, 5230 Odense M, Denmark

**Keywords:** Ceramide, Sphingosine-1-phosphate, Sphingolipids, Autophagy, Apoptosis, TOR

## Abstract

Maintenance of cellular homeostasis requires tight and coordinated control of numerous metabolic pathways, which are governed by interconnected networks of signaling pathways and energy-sensing regulators. Autophagy, a lysosomal degradation pathway by which the cell self-digests its own components, has over the past decade been recognized as an essential part of metabolism. Autophagy not only rids the cell of excessive or damaged organelles, misfolded proteins, and invading microorganisms, it also provides nutrients to maintain crucial cellular functions. Besides serving as essential structural moieties of biomembranes, lipids including sphingolipids are increasingly being recognized as central regulators of a number of important cellular processes, including autophagy. In the present review we describe how sphingolipids, with special emphasis on ceramides and sphingosine-1-phosphate, can act as physiological regulators of autophagy in relation to cellular and organismal growth, survival, and aging.

## Introduction

Autophagy is an intracellular degradation process, which is highly conserved among all eukaryotes. The term itself, meaning self-eating, refers to the fact that cells generate energy and cellular building blocks by degradation of its own components. In that sense autophagy is a pro-survival process, but in unfavorable situations autophagy can contribute to cell death [1]. Sphingolipids were once regarded as being merely structural elements in cell membranes. Now this diverse group of lipids is recognized as important mediators of cellular events, including autophagy, through their roles as functional entities in cellular membranes and as bioactive signaling molecules. Especially three sphingolipid species, ceramide, dihydroceramide (dhCer), and sphingosine-1-phosphate (S1P), have emerged as being important mediators of the autophagic pathway. They are believed to function as a rheostat controlling the balance between sphingolipid-induced autophagy and cell death [2]. Furthermore, sphingolipids are also important regulators of nutrient import from the extracellular environment and autophagic flux [3, 4]. This review aims to describe the interaction between autophagy and sphingolipid metabolism. Furthermore, we address the importance of subcellular localization of the sphingolipid species in this interplay.

## Autophagy-machinery and regulation

The term autophagy encompasses several different sub-processes classified according to how the cargo is transported to the lysosome, which include macroautophagy, microautophagy, and chaperone-mediated autophagy. Macroautophagy is the most prevalent form, and will accordingly be referred to as autophagy hereafter.

**Fig. 1:**
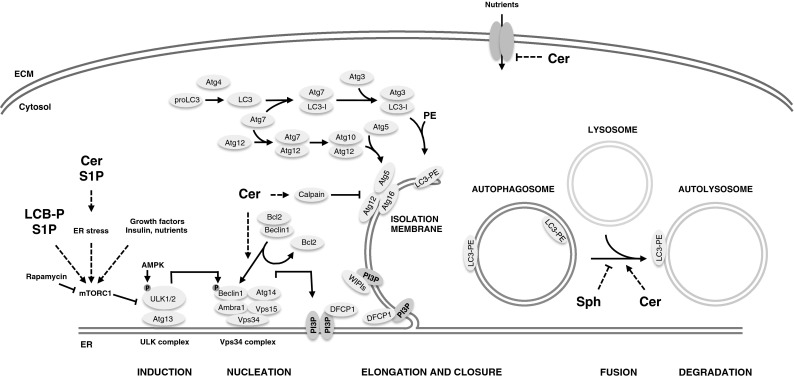
Ceramides and other sphingolipids regulate autophagy at multiple levels. mTOR complex 1 (mTORC1) phosphorylates and suppresses the ULK1 complex under nutrient-rich conditions. Upon induction of autophagy, the ULK1 complex is activated by AMP-activated protein kinase (AMPK) phosphorylation and by autophosphorylation to phosphorylate Beclin1, which promotes the formation of the Vps34/PI3-kinase complex and hence generation of phosphatidylinositol-3-phosphate (PI(3)P). This recruits PI(3)P-binding proteins like DFCP1 and WIPIs to the membrane and promotes the formation of autophagosomes. The Atg12–Atg5–Atg16 complex is required for conjugating phosphatidylethanolamine (PE) to LC3 for its attachment to the autophagosomes and hence for elongation and closure of the isolation membrane. Once complete, the outer membrane of the autophagosome fuses with the lysosome, and the material is degraded in the autolysosome by acidic hydrolases. Ceramides (Cer) have been shown to reduce the abundance of nutrient transporters in the plasma membrane resulting in lowered uptake of nutrients, hence activation of AMPK, suppression of TORC1 activity, and activation of autophagy. Ceramides also promote dissociation of the Bcl2–Beclin1 complex and affect ER homeostasis and fusion between autophagosomes and lysosomes. Moreover, ceramides have also been shown to affect calpain-mediated cleavage of Atg5. Sphingoid long-chain base phosphates have furthermore been shown to induce autophagy

The autophagic degradation pathway overall includes five levels: (1) The formation of a double-membrane structure (also denoted as the isolation membrane), (2) encapsulation of intracellular cargo, (3) formation of the mature autophagosome, (4) fusion with a lysosome, and (5) lysosomal degradation of the cargo (Fig. 1). Each of these critical steps are regulated and effected by a number of AuTophaGy-related proteins (the Atgs), first identified and described in yeast [5, 6], but since shown to have metazoan orthologs [7]. These specific proteins, some of which form complexes, each have their own role in the process of autophagy. The most prominent of these will be discussed briefly in the following section.

Without stimulation, autophagic activity is kept at a low basal level, but can be induced by extracellular cues such as stress, starvation, various pathologies, or by drug treatment. One very central gatekeeper of autophagy initiation is the target of rapamycin (TOR) complex, with the TOR kinase as a central catalytically active entity. The TOR kinase assembles into two structurally and functionally distinct complexes, referred to as TOR complex 1 (TORC1) and 2 (TORC2) [8]. If not inhibited by amino acid starvation, TORC1 inhibits the formation of the autophagosome at a very early step, namely the formation of the isolation membrane. By phosphorylation of Atg13, TORC1 prevents the formation of the Unc-51 like autophagy activating kinase 1 (ULK1) complex (Fig. 1). In times of amino acid starvation, TORC1 is inhibited [9] and ULK1 is free to phosphorylate Beclin-1, which in turn enhances the activity of VPS34, a class III phosphoinositide 3-kinase (PI3K) situated at the autophagosomal membrane. Hence, the formation of phosphatidylinositol-3-phosphate (PI(3)P) at the autophagosomal membrane is induced. This provides a platform for the gathering of autophagosomal actors and recruits PI(3)P-binding proteins like the Double FYVE-containing protein 1 (DFCP1) and WD-repeat protein interacting with phosphoinositides 1 and 2 (WIPI1 and WIPI2), which are both required for full induction of autophagy [10, 11].

In order for the isolation membrane to form and elongate, continuous recruitment and formation of cellular membrane structures are required. The endoplasmic reticulum (ER), Golgi, mitochondria, plasma membrane, lipid droplets, and ER-mitochondria contact sites have all been suggested to provide lipids for the formation of the autophagosomal isolation membrane [12–19]. The elongation of the growing pre-autophagosomal structure requires ubiquitination of Atg5 by the ubiquitin-like protein Atg12, which is activated by the E1-like enzyme Atg7 and the E2-like enzyme Atg10. The Atg12-Atg5 complex is subsequently linked to Atg16, forming a tetramer that dissociates from the mature autophagosome, which is required for the elongation process [20]. In a second ubiquitin-like reaction, in which the E1-like enzyme Atg7 and the E2-like enzyme Atg3 are involved, microtubule-associated protein 1A/1B-light chain 3 (LC3) is cleaved by Atg4 to form LC3-I, which is subsequently lipidated by the addition of phosphatidylethanolamine into LC3-II. LC3-II localizes both to the outer and inner autophagic membrane where it resides until degradation of the cargo and the inner autophagic membrane. Once the autophagosome is formed and matured, it can fuse with either an endosome to form an amphisome, which subsequently fuses with a lysosome, or directly with a lysosome to form an autolysosome. Although the precise molecular details underlying the fusion event still remain to be fully elucidated, it is known that the small GTP-binding protein named Rab7, the SNARE syntaxin Stx17, and components of the homotypic fusion and protein sorting (HOPS)-tethering complex serve as important factors in the autophagosome-lysosome fusion [21–23]. After the fusion and subsequent degradation of the autophagosomal cargo, the resulting material is transported back to the cytosol through lysosomal permeases, where it can be reused for energy production or as molecular building blocks [24].

Autophagy was originally considered to be a non-selective process, however during the past decade several studies have proven that autophagy can be highly selective. Indeed, proteins like nucleoporin p62 and Neighbor of BRCA1 gene 1 (NBR1) function as cargo receptors selectively collecting ubiquitinated substrates for autophagosomal degradation. p62 binds directly to LC3 and is degraded in parallel with the cargo, thus accumulating when degradation is inhibited. Recently, selective degradation of entire organelles such as lipid droplets, ER, mitochondria, and peroxisomes has been described [25], suggesting that autophagy can act specifically.

Whether autophagy is acting selectively or not, the regulation of this delicate process is highly critical. Within the past decade, it has become clear that sphingolipids serve as important regulators of this process at several levels.

## Sphingolipid metabolism

**Fig. 2:**
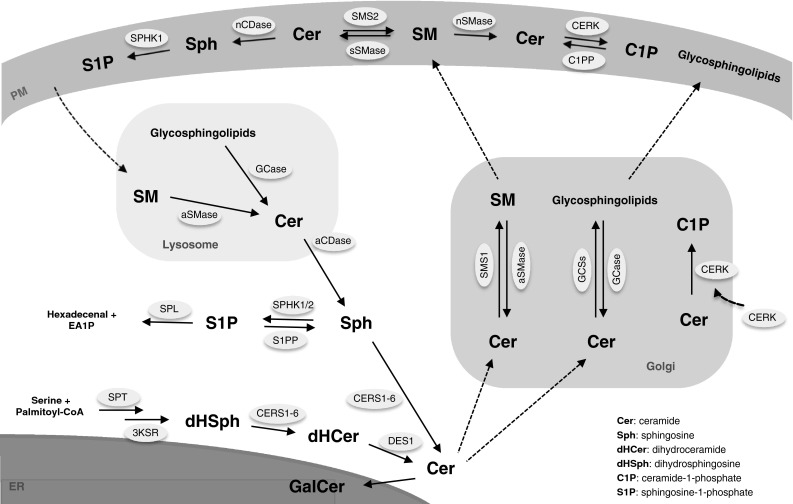
Overview of sphingolipid metabolism. Central in the sphingolipid metabolism is ceramide. Ceramide is de novo synthesized at the endoplasmatic reticulum (ER) with the condensation of serine and palmitoyl-CoA by serine palmitoyltransferase (SPT) being the first step. Further reduction and acylation by a ceramide synthase (CERS1-6) yields dihydrosphingosine (dHSph), which after desaturation results in the formation of ceramide. At the ER ceramide can be modified into galactosylceramide (GalCer), yet the majority of ceramide modification takes place at the Golgi in a manner depending on their further utilization. At the Golgi ceramide is used in the synthesis of sphingomyelin (SM) and glycosphingolipids in reactions catalyzed by sphingomyelin synthase 1 (SMS1) and glycosphingolipid synthases (GCSs), respectively. From the Golgi, SM and glycosphingolipids are transported to the plasma membrane (PM). Here SM can be turned into ceramide again by the actions of secretory and neutral sphingomyelinases (sSMase and nSMase, respectively). The ceramide can then be metabolized into ceramide-1-phosphate (C1P), sphingosine-1-phosphate (S1P), or be resynthesized back into SM. Complex sphingolipids residing in the PM can also be used as a pool for recycling of ceramide by entering the endolysosomal pathway. In this pathway acid SMase (aSMase) and glycosidases (GCase) produce ceramide, which in turn can be hydrolyzed into sphingosine and reused in the synthesis of ceramide or be degraded by phosphorylation into S1P followed by breakdown to hexadecenal and ethanolamine-1-phosphate (EA1P). In the Golgi, ceramide kinase (CERK) can phosphorylate ceramide thereby generating ceramide-1-phosphate (C1P). Other abbrevations: *3KSR* 3-ketosphinganine reductase, *CPP* ceramide phosphatase, *DES1* dihydroceramide desaturase 1, *aCDase* acid ceramidase, *nCDase* neutral ceramidase, *SPHK* sphingosine kinase, *aSMase* acid sphingomyelinase, *SPL* S1P lyase, *S1PP* sphingosine phosphate phosphatase

Sphingolipids comprise a large family of lipids, which differs structurally from other lipid species by containing a sphingoid base as structural backbone. Sphingolipid metabolism constitutes an interconnected network where balancing of sphingolipid synthesis, turnover, and recycling are carefully regulated according to cell response and fate. This network revolves around ceramide as depicted in Fig. 2. De novo synthesis of sphingolipids is initiated at the cytosolic face of the ER membrane, where serine palmitoyl-transferase (SPT) catalyzes the condensation of serine and palmitoyl-CoA producing 3-ketodihydrosphinganine. Next, 3-ketodihydrosphinganine is reduced to produce the sphingoid base dihydrosphingosine (sphinganine), which along with sphingosine, comprises the backbone of sphingolipids. Besides sphinganine the yeast *Saccharomyces cerevisiae* (*S. cerevisiae*) also produces phytosphingosine as a sphingoid base. Ceramide synthases (CERS1-6) catalyze the N-acylation of sphingoid bases resulting in synthesis of ceramides. The CERSs display different chain length specificities [26], which adds to the complexity of sphingolipids. CERS1 prefers C18-CoAs, while CERS2 utilizes acyl-CoAs ranging from C20 to C26. CERS3 shows preference towards the ultra-long-chain acyl-CoAs (C26–36), whereas CERS4 has specificity for C18- and C20-CoAs. CERS5 and CERS6 both primarily incorporate C16-CoAs. Ceramides can be phosphorylated to ceramide-1-phosphate (C1P), modified by addition of phosphocholine to yield sphingomyelin, or be glycosylated to produce a vast number of different glycosylceramides. Ceramides can be resynthesized from sphingosine or regenerated by recycling of glycosylceramides and sphingomyelin, or by dephosphorylation of C1P [27]. Importantly, while de novo synthesis of ceramides by CERSs requires hours [28], generation of ceramides from the recycling pathways, e.g. degradation of sphingomyelin, occurs within minutes of activation [29]. Thus, immediate regulation of cellular processes by ceramides and other sphingolipids may be mediated by salvaging pathways, whereas de novo synthesis of sphingolipids may modulate long-term cellular processes.

## Compartmentalization of sphingolipids is controlled by subcellular transport

The diversity of biomembranes is based on the vast structural heterogeneity of lipid species and their subcellular and asymmetric distribution. Such heterogeneity in organelles and membrane leaflets is critical in modulating the localization of membrane-bound proteins and their functional properties. Sphingolipids in the plasma membrane are normally exclusively found in the outer leaflet of the bilayer, and form together with cholesterol specific membrane microdomains [30, 31]. Such microdomains are laterally segregated regions, which arise as a result of selective affinities between sphingolipids and membrane proteins and presumably act as signaling platforms [31–34].

The distribution of sphingolipids in cellular organelles is far from uniform. It has become evident that sphingolipids are compartmentalized according to their functions, and that subcellular transport aids this sorting. The sorting initiates at the ER from where ceramide must be transported to the Golgi apparatus for further modification. Non-vesicular transport of ceramide requires the ceramide transfer protein (CERT) [35]. CERT has a preference for ceramide species containing acyl chains shorter than C22, while CERT-mediated transport of C22 and C24:1 ceramides, and of dhCer is significantly less efficient [36, 37]. Together with the fact that ceramides transported by CERT to the Golgi preferentially are incorporated into sphingomyelin compared to glycosphingolipids [38], this implies that CERT contributes to the complexity and subcellular distributions of specific sphingolipids. The restricted specificity of CERT also suggests that alternative routes for intracellular trafficking of ceramide must exist. Although not delineated in molecular detail yet, such alternative routes may include vesicular transport or be mediated through specific inter-organelle contact sites [39, 40].

Recently, a novel lipid transfer protein was identified by virtue of its ability to specifically transfer C1P between membranes, thus named C1P transfer protein (CPTP) [41]. Despite poor sequence homology, CPTP shares a very similar structural fold with glycolipid transfer protein (GLTP). However, it does not bind glycosylated ceramides like galactosylceramide and lactosylceramide as GLTP does. CPTP is localized in the cytosol, but it is also associated with the trans-Golgi network, nucleus, and plasma membrane, and has been proposed to control C1P levels to maintain proper Golgi organization and inflammatory responses [41].

Although it does not transport ceramides, the evolutionary conserved GLTP has been shown to accelerate transfer of both diacylglycerols- and sphingoid-based glycolipids between lipid membranes [42]. GLTP enables intermembrane transfer of glucosylceramide, lactosylceramide, galactosylceramide, sulfatide, and the gangliosides GM1 and GM3, but not phosphatidylcholine, phosphatidylethanolamine, sphingomyelin, phosphatidylinositol, cholesterol, and cholesterol oleate [43, 44]. Thus, although its function is yet to be fully resolved, it has been proposed that GLTP mediates the transfer of glycosylceramides from the Golgi to the plasma membrane or functions as an intracellular sensor of glycosphingolipids [45].

To this end, another member of the GLTP superfamily, the four-phosphate adaptor protein 2 (FAPP2) has also been shown to mediate intermembrane lipid transfer between the Golgi and the plasma membrane, and to be crucial for synthesis of complex glycosphingolipids in the Golgi network [46, 47]. The fact that cellular processes are highly compartmentalized too, paves the way for the idea of a very tight and local control of sphingolipid metabolism in relation to regulation of cellular processes.

## Sphingolipids and membrane fusion

Upon completion of the autophagic process, the autophagosome fuses with the lysosome to form the autolysosome, where sequestered organelles and proteins are degraded by acidic lysosomal hydrolases. The efficiency of this step depends on the cellular lipid composition, including the level of cholesterol and other lipids [48]. Even though sphingolipids have not directly been shown to affect fusion of autophagosomes with lysosomes, accumulating evidence suggests that this step also depends on sphingolipids. For example, it has recently been shown that myristate induces autophagy and autophagic flux in cardiomyocytes in a sphingolipid- and CERS5-dependent way, indicating that ceramide synthesis is involved in regulation of the autophagic flux [4]. Moreover, it has also been suggested that progression of autophagy in yeast depends on sphingolipid production. This is due to its role in formation of autophagosomes rather than conjugation of Atg12-Atg-5, lipidation of Atg8/LC3, maturation of vacuolar proteases, or the formation of the pre-autophagosomal structure [49].

It has previously been shown that the production of C1P from sphingomyelin, by the joint action of sphingomyelinase and ceramide kinase, promotes Ca^2+^-dependent liposomal fusion, which enhances the vesicle fusion. Interestingly, C1P levels increase during phagocytosis, indicating that C1P may promote fusion between phagosomes and lysosomes [50], and in turn signifying that C1P regulates autophagosome-to-lysosome fusion as well. Given the role of CPTP in controlling intracellular C1P levels [41], CPTP may also control such fusion events. Moreover, a recent study suggests a role for sphingosine kinase 1 (SPHK1) and its product, S1P, in the endo-and exocytotic membrane trafficking pathways [51], which are tightly linked to the autophagic pathway [52, 53].

The heterogeneity in organelles and membrane leaflets is, as previously mentioned, critical in modulating the localization of membrane-bound proteins and their functional properties. Changes in membrane lipid species can alter the membranes biophysical properties, which in turn affect biological properties. Ceramide can induce rigidization of membranes and hence may also affect the curvature of the membrane [54]. Accordingly, acid sphingomyelinase-induced synthesis of ceramide increases the packing of the lipids and is associated with enhanced order in membranes [55, 56]. It has been suggested that the hydrolysis of sphingomyelin to ceramide within the inner leaflet of the plasma membrane causes an outward curvature of the membrane important for exocytosis [57]. This function of membranous ceramide production may also apply to fusion of autophagosomes with lysosomes. By analogy, homotypic vacuole fusion, which shares a number of components with the fusion between autophagosomes and lysosomes, has been suggested to be affected by ceramide and other lipids in yeast [58, 59]. Moreover, sphingolipids have been shown to support fusion of enveloped animal viruses, such as Semliki Forest virus, with lipid bilayers [60, 61], arguing that sphingolipids are important in membrane fusion events.

In line with these studies, it has been found that an increase in sphingosine and a concomitant decrease in S1P are of pathological importance in the early events of the lysosomal storage disorder, Niemann-Pick type C1. The increase in sphingosine disturbs lysosomal Ca^2+^ homeostasis, subsequently blocking the late endosome-to-lysosome transport [62]. Moreover, accumulation of sphingomyelin is known to have a destabilizing effect on lysosomes [63] and to result in leakage of the lysosomal proteases to the cytosol [64]. A very recent study suggests that the increased levels of sphingomyelin observed in Niemann Pick disease type A, cause lysosomal dysfunction due to lysosomal membrane permeabilization [65]. This underlines that sphingolipids also function at another step of the lysosomal degradation pathway.

Collectively, these studies reveal an additional function of sphingolipids, besides their role as messenger molecules, in the control of the autophagic and lysosomal degradation pathways.

## The pollice verso of sphingolipids

**Fig. 3:**
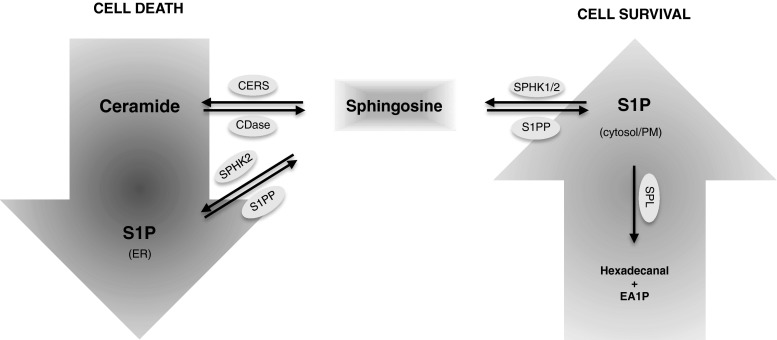
Sphingolipid-mediated regulation of cell death and survival. Regulation of the subcellular synthesis and localization of sphingolipids is crucial for their pro-apoptotic or pro-survival roles. Sphingosine kinase 2 (SPHK2)-dependent synthesis of sphingosine-1-phosphate (S1P) at the ER has been shown to promote apoptosis, while translocation of SPHK1 from the cytosol to the plasma membrane promotes synthesis of S1P at the plasma membrane and induces cell survival

The regulation of the delicate balance between proliferation and cell death is another important aspect where sphingolipids act as second messengers. Specifically, S1P and ceramide have proved important in the regulation of cell fate [66, 67], however, their effect on cell fate are very different [68, 69]. Both acting through autophagy, S1P is believed to promote cell survival and proliferation, whereas ceramide has been found to induce growth arrest and cell death [70]. These opposing roles of two so easily inter-convertible biomolecules have led to the manifestation of the sphingolipid rheostat [2], which describes the intimate balance between the intracellular levels of ceramide and S1P and its importance in regulating cell fate (Fig. 3). Furthermore, this sphingolipid rheostat is controlling cell fate, at least partly, by modulating autophagy [67]. As the conversion of ceramide to S1P only requires two steps [69, 71], the regulation of the enzymes balancing the concentrations and localizations of ceramide and S1P, is rather significant to cell fate.

In favor of cell survival, S1P has been observed as a critical player through its induction of autophagy [66]. S1P levels are balanced by their synthesis from sphingosine catalyzed by SPHKs, their dephosphorylation catalyzed by S1P phosphatases and phosphohydratases as well as their irreversible degradation catalyzed by S1P lyase (Fig. 2). Sphingosine, in turn, can be generated by hydrolysis of ceramide catalyzed by the ceramidase and removed by synthesis of ceramide by the action of the CERS. In agreement with this, knockdown of the ER-residing S1P phosphatase, the enzyme responsible for the degradation of S1P, augments S1P levels and induces autophagy [72].

Two distinct isoforms of SPHK have been characterized: SPHK1 and -2 sharing five conserved domains; however, their tissue distribution and developmental expression are distinct [73, 74]. SPHK1, which is cytosolic, is activated by several external stimuli to specifically signal growth and survival [69]. It has been recognized as a nutrient-sensitive regulator of autophagy [68], and starvation-induced autophagy is dependent on SPHK1 activity, a mechanism, which is conserved from yeast to mammals [68, 75]. Through its production of S1P, SPHK1 is therefore considered pro-survival, and accordingly SPHK1 has been shown to induce DNA transcription and cell proliferation [76].

It is not clear whether S1P signaling acts through or parallel to the mammalian TOR (mTOR) pathway in regulating autophagy [68, 72]. In a study by Lavieu et al. phosphorylation of two known downstream effectors of mTOR was found to be induced as response to overexpression of SPHK1 [68]. However, down-regulation of S1P phosphorylase-1, and thus the degradation of S1P, does not affect phosphorylation of mTOR nor its downstream effectors [72, 77]. Surprisingly, several studies have described S1P as an inhibitor of autophagy through activation of the mTOR pathway via specific S1P receptors in the plasma membrane [67, 78–80]. However, it is important to note that S1P signaling through cell surface receptors might differ from signaling within the cell.

The class III PI3K inhibitor, 3-methyladenine, which is known to prevent the formation of autophagosomes, does not affect S1P induced autophagy, whereas silencing of Atg5, a protein required at a later step in the autophagic process, does indeed inhibit S1P-induced autophagy [72]. This might either suggest that S1P is controlling the autophagic state through an entirely different signaling pathway, or that S1P is able to interact directly with the class III PI3K, preventing its inhibition by 3-methyladenine. In turn, the effect of S1P on cell survival is believed to be conducted through an inositol-independent Ca^2+^ mobilization from intracellular stores [81], activation of the mitogen activated protein pathway through the extracellular signal-regulated kinase ERK [82], and by suppression of the apoptotic effect of ceramide via inhibition of the stress-activated protein kinase c-Jun N-terminal protein kinase (JNK) [2].

Surprisingly, the other isoform of the kinase responsible for S1P production, SPHK2, is pro-apoptotic and has been found to localize in both the nucleus and cytosol, where it prevents DNA synthesis and cell proliferation, thus counteracting the positive effect of SPHK1 on cell division [83, 84]. Upon starvation SPHK2 is redirected to the ER, which is essential for its pro-apoptotic function [85]. Due to this re-localization, it has been suggested that SPHK2’s pro-apoptotic role is due to indirect production of ceramide from external sources of sphingoid bases by the combined actions of SPHK2 and lipid phosphohydrolases in the ER [69, 85]. Accordingly, down-regulation of SPHK2 reduces the conversion of sphingosine to ceramide via the recycling pathway, whereas down-regulation of SPHK1 increases it [85]. Interestingly, targeting of the pro-survival SPHK1 to the ER makes it pro-apoptotic [85], collectively suggesting that differently distributed intracellular pools of S1P might participate in different metabolic and signaling pathways. The death-inducing mitochondrial protein BCL2/adenovirus E1B 19 kDa protein-interacting protein 3 (BNIP3) is involved in ceramide-induced cell death and can displace Beclin 1 from the Beclin 1/Bcl-2 complex due to its Bcl-2 homology-3 (BH3) domain [86, 87]. The fact that SPHK2 also contains a BH3 domain that enables it to displace Beclin 1, supports the pro-apoptotic effect of SPHK2 [88]. In line with this, mutation of the BH3 domain causes suppression of the SPHK2 induced apoptosis [89]. Accordingly, it has been suggested that SPHK2 functions independently of its catalytic activity by production of S1P, but rather through its ability to interact with Bcl-2 [88].

Ceramide orchestrates programmed cell death through two different pathways referred to as type I and type II programmed cell death. Type I programmed cell death, apoptosis, is induced by increased ceramide levels [69]. The mechanisms for ceramide-induced apoptosis are numerous, including activation of caspase-9 through inactivation of protein kinase B (PKB), activation of Bad through Ras and protein phosphatase 2A (PP2A), dephosphorylation of retinoblastoma gene product by the activation of protein phosphatase 1, as well as activation of protein kinase Cζ and ceramide activated protein kinase [90–95].

Ceramide has not only been associated with apoptotic cell death, but also with another type of cell death distinct from apoptosis [96–98]. Whereas autophagy is normally associated with protection of the cell, an alternative strategy has been known for a decade, where cells consume their own interior resulting in a programmed cell death occurring independently of the apoptotic cell death [99]. This autophagic cell death is referred to as type II cell death [100] and is caspase-independent and characterized by a large number of autophagic vacuoles, early degradation of organelles, and preservation of cytoskeletal elements [100, 101], and by being independent of apoptosis and can be rescued by autophagic inhibition [1]. Furthermore, type II cell death can occur in the absence of the pro-apoptotic members of the Bcl-2 family [102]. It is activated by ceramide via induction of autophagy through lowering of the mitochondrial membrane potential, and by activation of the transcription of BNIP3 [98].

Whereas ceramide-induced autophagy and cell death act by activation of PP2A, thus inhibiting the pro-survival PKB [103], and subsequently augment Beclin 1 accumulation [1, 66], S1P promotes cell survival in a PKB-independent manner [103]. Since the anti-apoptotic protein Bcl-2 inhibits the function of Beclin 1 in the early stages of autophagy [104], the dissociation of the Beclin-1/Bcl-2 complex is required for induction of autophagy. Yet, ceramide has proved a potent inducer of this dissociation [105]. The release of Beclin 1 from the complex can be mediated by phosphorylation of Bcl-2 by the stress-activated JNK and is stimulated by supplementation of short-chain ceramides or by enhancing de novo synthesis of ceramide [105]. Accordingly, Beclin 1 mutation, which disables the protein to bind Bcl-2, induces autophagy resulting in cell death [104].

In contrast to the cell death-inducing effects of ceramide described above, Demarchi et al. found that C2-ceramide triggers an NF-кB dependent survival pathway, and that the induction of pro-survival genes is dependent on the protease calpain [106]. Calpain is induced upon starvation [107], inhibits apoptosis [108], and has been found to be required for autophagy as well as for the pro-survival effect of C2 ceramide [106, 109]. These opposing functions of ceramide have recently been coined the autophagy paradox [1], and have been suggested to function as a brake of the ceramide-induced cell death [106]. However, these observations may also be due to the artificial effects caused by the use of biologically irrelevant ceramides used in these studies.

In yeast TORC2 positively controls the synthesis of phytoceramide and dhCer [110]. Since ceramide, as described above, controls the induction of type II programmed cell death through activation of autophagy, it might seem rather conflicting that TORC2, which is otherwise known to support growth, activates the synthesis of ceramide. However, it is likely that phytoceramide and dhCers exert effects different from ceramides. Indeed, the action of ceramide and its analogs on cell death has been shown to be specific among the different lipid species [111]. The steady-state levels of sphingoid long-chain-bases and their phosphorylated derivatives in yeast were also shown to decrease in mutants lacking an ortholog of the mammalian TORC2 component Rictor [110]. This observation suggests that TORC2 might be involved in the production of S1P as well, which has been shown to reverse ceramide-mediated apoptosis [2]. In fact, the TORC2-dependent ceramide and S1P production might work as a feedback loop since both lipids are activators of autophagy, which is negatively regulated by TOR [68]. Since S1P is synthesized from recycling of ceramide [28], ceramide synthesis is required for production of this pro-survival second messenger. Interestingly, increasing levels of dhCer in response to inhibition of sphingolipid delta(4)-desaturase DES1 have been shown to delay the G1/S transition of the cell cycle in an autophagy-dependent manner, suggesting a pro-survival role for dhCer [112].

## Regulation of nutrient uptake by sphingolipids

Sphingolipids not only constitute significant structural entities in the plasma membrane, they also regulate the activity and abundance of nutrient transporters, thus indirectly affecting autophagy. Erdinger and co-workers showed that addition of C2-ceramides diminishes the surface abundance of the amino acid transferase, 4F2, resulting in impaired amino acid uptake, induced autophagy, and decreased viability [3]. Supplementation of methyl pyruvate, a membrane-permeant derivative of pyruvate, reversed C2-ceramide-induced cell death and autophagy independent of the surface abundance of 4F2, arguing that C2-ceramides cause starvation-induced cell death [3]. Similarly, Rosales et al. recently demonstrated that sphingolipid-based drugs down-regulate nutrient transporters, thereby inducing autophagy and killing cancer cells by nutrient deprivation [113]. Interestingly, inhibition of TORC1 in *S. cerevisiae* activates the nitrogen permease reactivator 1 kinase, which phosphorylates and hence relieves the inactivating effects of Orm1 and Orm2 on the SPT, resulting in increased de novo synthesis of complex sphingolipids. In turn, plasma membrane localization and activity of the general amino acid permease Gap1 is stimulated [114]. To this end, inhibition of SPT and complex sphingolipid synthesis have been shown to inhibit autophagy [49]. Moreover, when sphingolipid levels are low, the two phosphoinositide PI4,5P(2) binding proteins Slm1 and Slm2 recruit the kinases Ypk1 and Ypk2 to TORC2 at the plasma membrane, where they are phosphorylated and activated by TORC2 and the kinases Pkh1 and Pkh2 [115, 116]. Ypk1 and Ypk2 subsequently phosphorylate Orm1 and Orm2, which relieves their inhibition of SPT, thereby stimulating the synthesis of long-chain bases and sphingolipids [116, 117]. Interestingly, the ORMDL gene family encoding mammalian homologs of Orm1 and Orm2 has also been found to repress SPT activity and sphingolipid synthesis in mammalian cells in a phosphorylation-dependent manner [118], indicating that this regulatory mechanism is evolutionary conserved. These observations imply that nutrient uptake, TORC activities, and sphingolipid synthesis are coordinately regulated. Zimmerman et al. recently found that TORC1- and GSK3-dependent phosphorylation of Elo2 in *S. cerevisiae* promotes very-long chain fatty acid synthesis, while impaired phosphorylation results in a profound decrease in ceramide levels and a concomitant increase in the level of phosphorylated long-chain bases. The increase in phosphorylated long-chain bases resulted in constitutive induction of autophagy, which negatively affected cell viability, which again could be prevented by inactivation of the sphingoid long-chain base kinase Lcb4 [119].

Collectively, these observations suggest that import of nutrients via specific transporters and permeases is sensitive to changes in the sphingolipid level in the plasma membrane, which in turn is carefully controlled by networks of kinases and phosphatases in an auto-regulatory loop.

## Lifespan and sphingolipids

It is widely accepted that autophagy and lifespan are tightly linked [120–123]. Sphingolipids may therefore also affect organismal and cellular lifespan by modulating autophagy. Liu et al. have recently shown that both chemical- and genetic-inhibition of SPT activity increase lifespan in *S. cerevisiae* through reduced TORC1 activity and enhanced autophagy [124, 125], arguing that sphingolipids modulate lifespan. They also observed that lifespan extension induced by both calorie restriction and inhibition of S6 kinase was further augmented by myriocin in a dose-dependent manner [125], suggesting that impaired de novo sphingolipid synthesis induces longevity in parallel to caloric restriction and S6 kinase inhibition. Consistently, inhibition of TORC1 by rapamycin further enhanced myriocin-induced longevity, underlining that inhibition of sphingolipid synthesis synergistically with impaired TORC1 activity can extend life span [124, 125]. In line with this, inhibition of SPT1 activity in *Caernorhabditis elegans* (*C. elegans*) slowed the development rate and extended longevity [126]. Mosbech et al. recently found that impaired sphingolipid synthesis in *C. elegans*, caused by functional loss of the two ceramide synthases HYL-1 and LAGR-1, extended longevity in an autophagy-dependent manner [127]. Interestingly, loss of HYL-2 and LAGR-1 function had the opposite effect on lifespan, arguing that unique sphingolipid species and/or tissue-specific synthesis of sphingolipids are important in determining organismal longevity [127]. Moreover, *C. elegans* lacking ceramide glucosyl transferases arrests at the first larval stage which can be rescued by expression of ceramide glucosyl transferases in the most anterior- and posterior intestinal cells, implying that cell-specific synthesis of glycosphingolipids are indispensable for growth and survival [128]. In summary, lifespan and development are under the control of sphingolipid metabolism, possibly through regulation of autophagy.

## Concluding remarks

Sphingolipids comprise a diverse group of lipid species, which are highly interchangeable, and therefore constitute an ideal second messenger. The exact mechanisms governing sphingolipid-mediated regulation of autophagy and other cellular processes still remain enigmatic; however, their roles in autophagy appear to be mediated at different stages of the autophagic process. Sphingolipids constitute a major part of the plasma membrane and by virtue of their biophysical properties and interactions with membrane proteins, sphingolipids and proteins cluster to form microdomains with unique regulatory functions. Alterations in membrane sphingolipids can modulate the level and activity of nutrient transporters [3, 113, 129, 130] and therefore impair nutrient uptake and indirectly induce autophagy. Sphingolipids may also affect autophagy by regulating the assembly of the autophagic machinery, and affect fusion between autophagosomes and lysosomes, either by modulating the membrane properties, abundance of fusogenic SNARE proteins, or acidification of the lysosomes [48, 58, 59, 131]. However, since specific sphingolipids like S1P modulate particular cellular processes, while other closely related species may exert opposite effects [54, 132, 133], the enzymes responsible for their interconversions serve central roles in regulation of cellular metabolism and cell fate. It is therefore also critical that the localization of sphingolipids in specific cellular compartments is tightly controlled. Thus, intracellular transport of sphingolipids by transfer proteins like CERT, CPTP, FAPP2, and GLTP, is crucial for their regulatory properties.

Considering the regulatory roles of sphingolipids in autophagy, it is interesting that autophagy regulates sphingolipid levels including ceramide levels [134] and mobilization as well as storage of glycerolipids in lipid droplets [19, 135, 136]. Collectively, this shows that autophagy and lipid metabolism are coordinately controlled, and underlines the importance of sphingolipids in metabolic regulation. However, some of the reported effects of sphingolipids are based on short-chain ceramides, which may have completely different effects from ceramides produced in vivo. Therefore, rather than using such non-natural ceramides, the roles of sphingolipids in autophagy should be examined further via loss-of-function and overexpression studies in genetically tractable model systems. Such studies will broaden our understanding of how sphingolipids affect specific stages of autophagy.

## Abbreviations

Atg: Autophagy-related
BH3: Bcl-2 homology-3
BNIP3: BCL2/adenovirus E1B 19 kDa protein-interacting protein 3
*C. elegans*: *Caernorhabditis elegans*
C1P: Ceramide-1-phosphate
CERT: Ceramide transfer protein
CERS: Ceramide synthase
CPTP: Ceramide-1-phosphate transfer protein
dhCer: Dihydroceramide
DFCP1: Double FYVE-containing protein 1
ER: Endoplasmic reticulum
FAPP2: Four-phosphate adaptor protein 2
GLTP: Glycolipid transfer protein
JNK: c-Jun N-terminal protein kinase
LC3: Microtubule-associated protein 1 light chain 3
mTOR: Mammalian target of rapamycin
NBR1: Neighbor of BRCA1 gene 1
PI3 K: Phosphoinositide 3-kinase
PI(3)P: Phosphatidylinositol-3-phosphate
PKB: Protein kinase B
PP2A: Protein phosphatase 2A
*S. cerevisiae*: *Saccharomyces cerevisiae*
SPT: Serine palmitoyltransferase
SPHK: Sphingosine kinase
S1P: Sphingosine-1-phosphate
TOR: Target of rapamycin
TORC: TOR complex
ULK1: Unc-51 like autophagy activating kinase 1
WIPI: WD-repeat protein interacting with phosphoinosides
